# Accreditation of HIV services to improve infection control system of hospital – experience from India

**DOI:** 10.1186/2047-2994-4-S1-P113

**Published:** 2015-06-16

**Authors:** R Lalthanmawia

**Affiliations:** 1 Community Health Department, Christian Medical Association of India, New Delhi, India

## Introduction

Patient care in hospital has been assessed with various tools like PLHIV Friendly Checklist for Hospital developed by UNAIDS. These process helps in acknowledging the constraints within hospital to provide stigma and discrimination free services to PLHIV even in general hospitals. In order to ensure that quality standards are maintained, Christian Medical Association of India developed an Accreditation process by which the member institutions are assessed at various interval of times.

## Objectives

To ensure strengthening of infection control for patient safety through an Accreditation of HIV Services.

## Methods

Christian Medical Association of India (CMAI) as a network of Christian Hospitals in India developed an Accreditation tools with inputs from experts to assess the quality of HIV Services provided in the hospitals. CMAI trained and oriented experts in the field to conduct the assessment in selected hospitals. The results were discussed with the hospital and action plans for improvement were made in consultation.

## Results

CMAI assessed 5 of the member institutions as a pilot to assess the services provided by the hospital to be PLHIV friendly. The areas assessed included Counseling, Out – patient HIV care, Medical Care, Obstetric Care, Surgical Care, HIV Testing, Infection Control, Blood Safety, Post - Exposure Prophylaxis, Staff Education, HIV Team, HIV Policy and guidelines, Organization of positive people/Community Organization, Networking with NGO/s private practitioners and other hospitals, Home based care, Discrimination/Stigma, Drug availability and access. A total of 105 were given with each component having different levels of quality improvement. The hospitals assessed scored 67, 85, 73, 102 and 59. It was observed that areas like networking and support groups were lacking in most of the hospital. Policy and guidelines to ensure implementation of the stigma–free and non–discriminatory environment was also a necessity.

## Conclusion

A comprehensive care and support for PLHIV extends beyond hospital settings and needs to be a part of the service provided in a hospital with necessary linkages and networking. Accreditation of HIV services assisted the hospital in developing action plans for improvement and also monitor accordingly.

## Disclosure of interest

None declared.

